# Analyzing the barriers and enablers to internet hospital implementation: a qualitative study of a tertiary hospital using TDF and COM-B framework

**DOI:** 10.3389/fdgth.2024.1362395

**Published:** 2024-08-08

**Authors:** Xiaolong Wu, Yulin Kuang, Yonglin Guo, Ning Wei, Zichun Fan, Jingru Ling

**Affiliations:** ^1^ Business School, Sun Yat-sen University, Shenzhen, Guangdong, China; ^2^ School of Government, Sun Yat-sen University, Shenzhen, Guangdong, China; ^3^ Shenzhen Maternity & Child Healthcare Hospital, Shenzhen, Guangdong, China

**Keywords:** Internet hospitals, qualitative study, theoretical domains framework, COM-B model, e-Health services, motivation

## Abstract

**Background:**

Internet hospitals have become an important way to improve the accessibility of medical services and promote medical equity in China. However, there is still lack of research on the behavior of medical personnel during the process of using Internet medical services, and the elements of behavior that motivate doctors to actively use or resist the use of Internet hospitals are still not fully analyzed. The study applied the Theoretical Domains Framework to examine the factors affecting the engagement of medical personnel in Internet hospitals, with the aim of guiding the design of intervention to enhance Internet hospital participation.

**Methods:**

This study utilized qualitative analysis. Semi-structured questionnaires based on the Theoretical Domains Framework (TDF) and Capability-Opportunity-Motivation-Behavior (COM-B) model was developed and administered to 40 doctors and nurses at a Grade A tertiary hospital in Guangdong Province. Data was coded and analyzed using qualitative methods including Nvivo software.

**Results:**

The research displayed 19 barriers and 7 enablers for the implementation of Internet hospitals, all 14 TDF domains impacted participation with motivation cited most frequently. Despite challenges, medical personnel exhibited a generally optimistic stance towards utilization of the Internet hospital. Major barriers include the higher requirement of diagnostic ability, objective difficulties brought by online consultation to the decision-making process, limitation of time and other resources, not ideal technological and institutional environment, lack of self-efficacy and negative expectation of results in online consultation. Key enablers include patient needs and the positive impact of online care on the medical process and patient experience.

**Discussion:**

This qualitative study identified a range of barriers and enablers to Internet hospital participation according to medical personnel, providing an conceptual framework to guide further research evaluating implementation strategies. Expanded research and targeted interventions design can help optimize participation in this evolving healthcare delivery model.

## 1 Introduction

Internet hospitals have emerged as a new way for doctors to provide healthcare services and education using telecommunications technology. This enables doctors to conduct diagnosis and treatment activities online, and provide subsequent visiting services such as online consultation, prescribing and dispensing for patients diagnosed with common or chronic diseases in offline hospitals (1). The main purpose of introducing Internet hospitals is to reduce the difficulty in accessing modern healthcare for rural populations (2). The COVID-19 pandemic has further accelerated the development of Internet hospitals, as mandated restrictions and widespread lockdowns have disrupted routine patient treatments for other diseases. Online medical support during the outbreak has reduced social panic, promoted social distancing, and reduced the chance of cross-infection, playing an important role in preventing and controlling COVID-19 (3). As a result, there has been a significant increase in visits to Internet outpatient clinics, which has considerably boosted the medical service capacity of Internet hospitals (4–6).

### 1.1 The effectiveness of the internet hospital

As Internet hospitals continue to develop, opinions on their effectiveness are varied. On one hand, they have provided patients who face geographical or time constraints with convenient access to medical services. Patients can easily consult doctors online from the comfort of their homes, saving time and reducing costs (7, 8). However, some have expressed concerns that doctors may lack sufficient information to make accurate diagnoses or provide appropriate treatment without access to the patient's complete medical history. Another advantage of Internet hospitals is that they improve medical efficiency by reducing patient wait times and alleviating the workload of doctors in traditional hospitals, thus also addressing the shortage of medical resources in China (9). On the other hand, doctors may experience a heavier workload as they have to respond to a large number of online consultations without access to patient background information (10). This can lead to burnout and a decline in the quality of services (9). While some argue that Internet hospitals have enhanced the doctor-patient relationship by providing personalized and timely medical services (11), others believe that the lack of face-to-face interaction may result in a weakening of emotional connections between doctors and their patients (12, 13).

Overall, it is clear that different entities hold varying views on the effectiveness of Internet hospitals. While Internet hospitals have made significant progress, they still face numerous challenges and need to improve their provision of health services (14, 15). Existing literature has showed some understanding that implementing Internet hospitals can result in both desirable and undesirable outcomes, but research on barriers to and enablers of Internet hospital implementation remains limited and scattered. Therefore, it is necessary to identify the barriers and enablers to the use of Internet hospitals from a more theoretical perspective.

### 1.2 Theoretical framework

This paper analyzes the comments of Internet hospitals by categorizing them into 14 domains in the Theoretical Domains Framework (TDF) and mapping them into their associated Capability-Opportunity-Motivation-Behavior (COM-B) model. Interventions based on theoretical models of behavior have been shown to be more effective than non-theoretical interventions, so it is necessary for us to incorporate theory into the design of behavior change strategies (16). The COM-B model and TDF have been widely used in studies to assess behavioral barriers and facilitators and to inform targeted interventions, so we take it as the theoretical framework of this paper.

The TDF encompasses 33 theories of behavior and behavior change across 14 domains, providing a theoretical perspective to understand the cognitive, affective, social, and environmental influences on individual and collective behavior resulting from the implementation of new practices in organizations, services, and systems. It offers a theoretical basis for implementation studies and good coverage of potential reasons for the slow diffusion of evidence into practice, as well as a method for progressing from theory-based investigation to intervention (17).

The COM-B model consolidates the 14 domains in the TDF into three principal domains that intertwine to understand and predict behavior, encompassing individuals' capacity(C), opportunity(O), and motivation(M) conducive to the behavior. The COM-B model of behavior is widely used to identify what needs to change for a behavior change intervention to be effective. Capability refers to an individual's psychological and physical ability to engage in an activity, opportunity refers to external factors that make behavior possible, and motivation refers to the conscious and unconscious cognitive processes that guide and inspire behavior (18). The COM-B model identifies three factors that need to be present for any behavior: capability, opportunity, and motivation. These factors interact over time, so behavior can be seen as part of a dynamic system with positive and negative feedback loops.

Overall, this approach provides a comprehensive framework for understanding the factors that contribute to behavior change in the context of Internet hospitals. By identifying areas where interventions can be implemented, healthcare providers can optimize patient outcomes and improve the overall effectiveness of Internet hospitals.

### 1.3 Significance and objective

Consequently, the aim of this study is to answer the question: “What are the barriers and enablers to Internet hospital implementation?” Our approach involves obtaining more systematic results on the barriers and enablers to Internet hospital adoption from the perspectives of doctors and nurses, using TDF and COM-B frameworks. We aim to identify the primary influencing factors to explore strategies for the development of Internet hospital platforms. By utilizing the TDF and COM-B frameworks, we aim to provide a more comprehensive understanding of the factors influencing the implementation of Internet hospitals and to identify potential areas of intervention. This study will not only shed light on the barriers and enablers to Internet hospital adoption, but it will also contribute to the overall effectiveness and efficiency of healthcare systems.

## 2 Method

This study utilized qualitative analysis to collect and analyze data from questionnaires based on the TDF. The data was coded and analyzed using Nvivo software. This study is reported according to the consolidated criteria for reporting qualitative research (19) (See Supplementary File 1). Informed consent was obtained from all interviewees before the study, and the the Ethical Committee of Shenzhen Maternal and Child Health Hospital has been consulted before submission of this paper, and ethical approval of this study is not required.

### 2.1 Participants

This study was conducted from March to April in 2023 at a Grade A tertiary hospital in Guangdong Province, China, which represents a high level of medical standards and professionalism, ideal for serving the objectives of this research. The corresponding author, affiliated with this hospital, facilitated the understanding and participation of the hospital's medical staff in our study. The study used purposive sampling and we selected a diverse cohort of 25 doctors and nurses eager to participate, based on their years of service, ages, departments, specialties, professional designations, and offline monthly outpatient diagnosis and treatment caseloads. This diversity guaranteed a comprehensive representation of professional perspectives. Additionally, our selection encompassed individuals who have utilized Internet hospital services, as well as those who have not, as we believe this approach not only captures authentic experiences with Internet hospitals, but also allows for a more thorough understanding of the current shortcomings in Internet hospital infrastructure, further aiding in the development of intervention strategies. The first round of survey was during the period of high incidence of Omicron virus, and the clinical work pressure of primary medical staff was great, so there were few medical personnel who had the willingness and time to participate in the survey. Therefore, we conducted a supplementary survey on another tertiary hospital in Shenzhen in the same way in March 2024 to check whether the new information appear, judging whether the data we obtained previously is robust.

### 2.2 The survey procedure

In accordance with the TDF and COM-B model, we formulated a questionnaire comprising 46 items of structured and semi-structured inquiries intended to collect detailed insights on personal details, usage experiences, perceptions, and an open section for participants to share additional perspectives (see Table 1 for the questionnaire overview). Questions were designed to assess doctors' and nurses' capabilities, opportunities, and motivations related to the adoption of Internet hospital services. This inclusion allowed us to gain nuanced insights into both the barriers and enablers inherent to the implementation of Internet hospitals. After the consent of the respondents, the survey were conducted through a secure online interview platform to uphold the integrity and confidentiality of the responses. The response rate was 100%, as all 25 in the first round and 15 in the second round invited medical personnel completed the survey.

**Table 1:** The questionnaire outline.

Attributes	Topics	
Personal information	Name, age, department, specialties, years of working, professional title	
Whether to provide relevant services/use relevant technology/carry out relevant work and how	Yes	1. How to obtain it 2. The reasons & How to organize and promote 3. The process 4. Personal Specific tasks & How was it accomplished & Memorable experiences 5. The degree of difficulty to provide/use/carry out
	No	1. The intended time to do 2. The preparations
Significance/goals	1. The opinions on the effectiveness of Internet hospitals 2. Whether it worked to achieve your goals and why	
Negative impacts	\	
Comparative advantages and disadvantages with traditional modes	\	
Similarities and differences with traditional modes	\	
The influencing factors of the implementation	Capabilities	What capabilities are needed (knowledge/skills/psychological capabilities)
	Opportunities	1. The impacts of time and resources 2. How to view the technology itself and the usage process 3. How to use it in contact with objects 4. How others influence the practice process
	Motivations	1. The feelings about using it or not using it 2. Do you consider using it beneficial and why
How to evaluate your performance	\	
Current difficulties	1. The solutions you can think of 2. What resources and support could assist you	

### 2.3 Data analysis

The data were collected and entered into an Excel database. Nvivo, a powerful software for qualitative analysis, was used for data coding and analysis after we confirmed that the information obtained is sufficient to analyze our own research objectives. Considering the reflexibility of the research, multiple team members with diverse professional backgrounds reviewed the data independently to ensure a balanced interpretation, mitigateing this problem.

To analyze the data, we carried out structured analysis, coding the data, involving inductive abstraction of main categories from preliminary codes, and establishment of links between categories and main categories. Each response was mapped to relevant domains within the TDF, and subsequently to the components of the COM-B model, thereby ensuring a comprehensive evaluation of factors influencing Internet hospital adoption. We also checked for whether there is any emerging theme which might suggest the need for additional context-specific categories, but in our analysis, we found that the existing domains sufficiently covered the relevant factors without requiring modifications. So the analyzed data were categorized into 14 domains within TDF, and these domains were further categorized into three larger domains in COM-B model.

Overall, this methodology provides a complete framework for analyzing the data collected from healthcare professionals regarding the barriers and enablers of Internet hospital adoption. The use of a comprehensive questionnaire and powerful software for qualitative analysis ensures that the data is accurately collected, coded, and analyzed, providing valuable insights for the development of Internet hospital platforms.

Statement: the Ethical Committee of Shenzhen Maternal and Child Health Hospital has been consulted before submission of this paper, and ethical approval of this study is not required.

## 3 Result

### 3.1 Participant characteristics

A total of 25 clinicians and nurses from different clinical and administrative departments participated in our research in the first round, and the number of 15 medical personnel participated in the second round. Participant characteristics are summarized in Table 2.

**Table 2:** Summary of participants’ characteristics.

The first round survey (Size: 25)	
Age (mean: 37.24)	
20–30	4
31–40	14
41–50	5
51–60	2
Years of practice (mean: 12.68)	
0–10	11
11–20	11
>20	3
Category of department	
Clinical departments (18)	Obstetrics and Gynecology (5/18)
	Pediatrics (5/18)
	Otolaryngology (1/18)
	Emergency (1/18)
	Reproductive Medicine (1/18)
	Nutrition (1/18)
	Acupuncture and Tuina (1/18)
	Traditional Chinese Medicine (2/18)
	Dermatology (1/18)
Health care department (3)	Children's Psychology and Rehabilitation (2/3)
	Women's Health (1/3)
Medical technology department (3)	Clinical Laboratory (1/3)
	Pathology (1/3)
	Ultrasound (1/3)
Administrative department (1)	Administrative Department (1/1)
Professional title	
Professor of medicine	3
Associate doctor	5
Attending doctor	10
Resident doctor	3
Deputy nurse-in-charge	1
Nurse-in-charge	3
Whether to participate in the Internet online consultation platform	
Yes	13
No	12
The second round survey (size: 15)	
Age (mean: 35.20)	
31–40	14
41–50	1
Years of practice (mean: 7.6)	
0–10	12
11–20	3
Category of department	
Clinical departments (11)	Neurology (1/11)
	Urology (1/11)
	Otolaryngology (1/11)
	Obstetrics and Gynecology (2/11)
	Cardiology (1/11)
	Reproductive Medicine (2/11)
	Endocrinology (2/11)
	Nephropathy (1/11)
Health care department (1)	Children's Psychology and Rehabilitation (1/1)
Medical technology department (2)	Nuclear Medicine Department (1)
	Information Department (1)
Administrative department (1)	Administrative Department (1/1)
Professional title	
Associate doctor	12
Attending doctor	3
Whether to participate in the Internet online consultation platform	
Yes	12
No	3

### 3.2 Factors influencing internet hospital implementation

**Figure 1:**
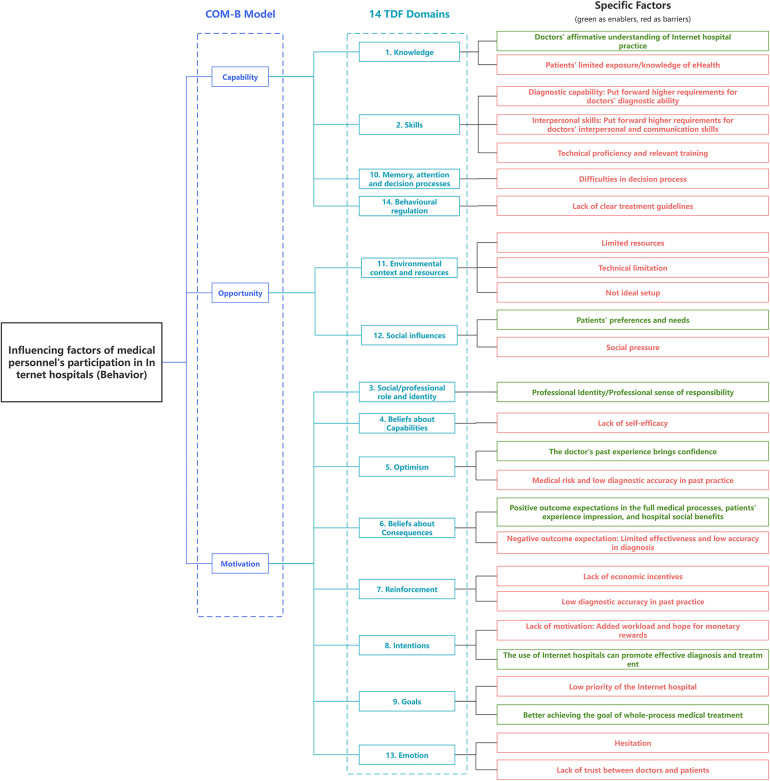
Visualization of survey results in COM-B and TDF frameworks.

We finished the data analysis after the first round survey, the obtained data was encoded and mapped to the TDF domain and COM-B model, and a coding tree was formed: the first-level coding refers to the three elements in the COM-B model, the second-level coding includes the 14 domains of the TDF framework, and the third-level coding includes 26 themes summarized by the questionnaire survey. In addition to topics derived from structural problems, each theme is supported by representative quotes (Table 3) (17, 20). This visualization of the result is also shown in Figure 1. The encoding of the TDF domain is the same as that formulated by the framework itself, all the themes are mapped to at least one of the 14 domains of the TDF and one of the components of COM-B model, and all TDF domains are mapped to. The second round survey is conducted after the data had already been analysed, aiming to verify the robustness of the research result based on the first round survey. After we processing the second round survey, we compared the collected new information with the results of the first round of analysis, and found that the second round of survey did not add new third-level coding in any domain, indicating that the first round of survey had been robust and basically reached the information saturation goal of qualitative research.

**Table 3:** Table of findings by TDF domains and COM-B model (17, 20).

Domains	Themes (barriers/enablers)	References
Capability (34.00%)		
1. Knowledge: What doctors and patients know on Internet hospital diagnosis and treatment.	Doctors’ affirmative understanding of Internet hospital practice (E)	"The Internet hospital is a progressive product that can facilitate patients’ treatment……"
	Patients’ limited exposure/knowledge of eHealth (B)	More than half of the participants said that patients’ view that in-person medical care is superior to telemedicine is an important reason that hinders medical staff from participating in Internet hospital diagnosis and treatment.
2. Skills: What doctors know about how they should perform on the Internet hospital.	Diagnostic capability: Put forward higher requirements for doctors’ diagnostic ability (B)	"I need to complete the diagnosis and treatment tasks independently without the guidance of a superior doctor.” “Because we can't diagnosis face to face, we need to make a treatment plan under the situation that the medical history and examination are not very clear."
	Interpersonal skills: Put forward higher requirements for doctors’ interpersonal and communication skills (B)	"Online diagnosis and treatment is mostly discontinuous, the delay time is long, the family expression is often lengthy, consuming the patience of medical staff."
	Technical proficiency and relevant training (B)	"Poor application of technology will affect work efficiency."
10. Memory, attention and decision processes: Decision process regarding Internet hospital diagnosis and treatment.	Difficulties in decision process (B)	"Due to resource constraints and inability to check the body, there is an evaluation bias."
14. Behavioural regulation: Management of doctors’ diagnosis and treatment behavior in Internet hospitals.	Lack of clear treatment guidelines (B)	"At present, there is a lack of specific guidelines for Internet hospital diagnosis and treatment."
Opportunity (14.81%)		
11. Environmental context and resources: Influence of the environment on doctors’ behaviour.	Limited resources (B)	"Doctors have limited time and energy, and discontinuous communication can eat into rest time."
	Technical limitation (B)	"Network speed affects the effectiveness of Internet diagnosis and treatment."
	Not ideal setup (B)	"If the Internet hospital system and mechanism is more sound and can effectively protect the safety of doctors, nurses and patients… I support the launch of the Internet hospital."
12. Social influences: How others influence doctors’ behaviour of Internet diagnosis and treatment.	Patients’ preferences and needs (E)	"It is easier for people to see a doctor and consult a doctor, and they can visit a specialist without limitation by geography."
	Social pressure(B)	"Many parents are prone to anxiety and expect complete satisfaction without seeing the patient."
Motivation (51.19%)		
3. Social/professional role and identity: Personal qualities such as professional attitude and responsibility of medical personnel.	Professional Identity/Professional sense of responsibility (E)	"I have a sense of responsibility and care for the patients in the Internet hospital."
4. Beliefs about capabilities: Perceived capability of doctors to perform Internet diagnosis and treatment.	Lack of self-efficacy (B)	"The psychological experience of doctors in online diagnosis and treatment is different from that offline: Online consultation is relatively uncertain so I may feel unsure about my diagnosis."
5. Optimism: The confidence of medical personnel in the outcomes that can be achieved by participating in Internet hospitals.	The doctor's past experience brings confidence (E)	"Personally, I made inquiries in some public accounts and pay different prices for different doctors, they all listened carefully and answered questions in detail, rather than copied and pasted the textbook for you, so that you feel the money is well spent."
	Medical risk and low diagnostic accuracy in past practice (B)	"At present, the rate of misdiagnosis and missed diagnosis on the Internet consultation is high."
6. Beliefs about Consequences: The doctors’ opinion about what could happen from performing Internet diagnosis and treatment.	Positive outcome expectations in the full medical processes, patients’ experience impression, and hospital social benefits (E)	"Avoid some face to face embarrassment (for example, some diseases are difficult for patients to talk about), reduce traffic jams around the hospital, and avoid unnecessary second visit to the hospital for prescribing the same medicine."
	Negative outcome expectation: Limited effectiveness and low accuracy in diagnosis (B)	"It is not conducive to physical examination and family guidance, because rehabilitation professional needs a lot of physical examination, and the family guidance (of rehabilitation movements) sometimes need to be taught face-to-face."
7. Reinforcement: Measures to stimulate the willingness of medical personnel to participate.	Lack of economic incentives (B)	"Not charging allows some people to waste resources excessively."
	Low diagnostic accuracy in past practice (B)	"Online diagnosis will not be too accurate, and the rate of misdiagnosis and missed diagnosis is high."
8. Intentions: The inclination of doctors to practice Internet diagnosis and treatment.	Lack of motivation: Added workload and hope for monetary rewards (B)	"Internet diagnosis and treatment takes more time and should be paid economically."
	The use of Internet hospitals can promote effective diagnosis and treatment (E)	"I can know the situation of patients in advance, which is conducive to the return visit and save time."
9. Goals: How important is Internet hospital practice for stakeholders.	Low priority of the Internet hospital (B)	"Internet hospitals have been basically suspended due to excessive use of personal time, inefficient communication and high medical risks."
	Better achieving the goal of whole-process medical treatment (E)	"Using the Internet makes it easy to answer patient consultations and follow up regularly."
13. Emotion: How doctors feel during the Internet hospital process.	Hesitation (B)	"Due to the uncertainty of online consultation, the psychological feelings of doctors in online diagnosis and treatment are different from offline, and they will be mentally uncertain and hesitation."
	Lack of trust between doctors and patients (B)	"The process of communication lacks emotional support, and it is difficult to establish a sense of trust between doctors and patients in a short time."

#### 3.2.1 Capability

Seven themes including four TDF domains (Knowledge; Skills; Memory, Attention and Decision Processes; Behavioral regulation) relate to capability component. The mentions of these seven themes accounted for 34.00% of the total.

##### 3.2.1.1 Knowledge

Some participants expressed affirmation of Internet hospital, and their positive understanding promoted their participation in the Internet hospital.

*“The Internet hospital is a progressive product that can facilitate patients’ treatment……”*

However, we know from participants that the main reason why they do not participate in the Internet hospital is because, some patients' disagreement with the effect of online treatment in their subjective willingness and refuse to use it. It follows that patient's limited knowledge of eHealth's effectiveness is a barrier of Internet hospital implementation.

##### 3.2.1.2 Skills

The characteristics of online consultation in Internet hospitals determine that doctors need to complete diagnosis independently without access to patients, which puts forward higher requirements for professional ability.

*“I need to complete the diagnosis and treatment tasks independently without the guidance of a superior doctor.”*

*"Because we can't diagnosis face to face, we need to make a treatment plan under the situation that the medical history and examination are not very clear.”*

Mental abilities like interpersonal skills and emotional intelligence are critical for effective diagnosis and treatment (21). Patience improves doctor-patient relationships.

*"Online diagnosis and treatment is mostly discontinuous, the delay time is long, the expression is often lengthy, consuming the patience of medical staff.”*

As relatively new technology, technical proficiency and training will impact use experience and effect. Adequate training is required for competence, but over 50% of respondents felt insufficiently trained.

*“Poor application of technology will affect work efficiency.”*

##### 3.2.1.3 Memory, attention and decision processes

Lack of examination, incomplete acquisition of medical history and other information, and discontinuous and incomplete communication of diseases make it difficult to obtain complete and sufficient information, which brings challenges to the diagnosis process.

*“Due to resource constraints and inability to check the body, there is an evaluation bias.”*

##### 3.2.1.4 Behavioral regulation

Clinical practice guidelines exist widely in all medical fields and help to form normative medical behavior. A participant indicated a need for a practice guidelines.

*“At present, there is a lack of specific guidelines for Internet hospital diagnosis and treatment.”*

#### 3.2.2 Opportunity

Five themes including two TDF domains (Environmental context and resources; Social influences) relate to opportunity component. The mentions of these six themes accounted for 14.81% of the total.

##### 3.2.2.1 Environmental context and resources

Participants noted resource constraints, mostly time, made effective implementation of Internet-based clinics difficult. Technical restrictions prevent effective Internet treatment, reducing diagnostic efficacy.

*“Doctors have limited time and energy, and discontinuous communication can eat into rest time.”*

*“Network speed affects the effectiveness of Internet diagnosis and treatment.”*

Imperfect system processes and lack of external support (e.g., legal protection and evaluation mechanisms) hinder Internet hospitals from achieving ideal operating environments, reducing participation.

*"If the Internet hospital system and mechanism is more sound and can effectively protect the safety of doctors, nurses and patients… I support the launch of the Internet hospital.”*

##### 3.2.2.2 Social influences

The advantages of Internet hospitals can indeed meet the specific needs or preferences of patients in practice, and provide a good social environment for the development of Internet hospitals.

*“It is easier for people to see a doctor and consult a doctor, and they can visit a specialist without limitation by geography.”*

The high demands and expectations of some patients and their families have brought social pressure virtually to doctors and nurses.

*“Many parents are prone to anxiety and expect complete satisfaction of medical service without without the doctor ever seeing the patient.”*

#### 3.2.3 Motivation

Fourteen themes including eight TDF domains (Social/professional role and identity; Beliefs about capabilities; Optimism; Beliefs about consequences; Reinforcement; Goals; Emotion) relate to motivation component. The mentions of these fourteen themes accounted for 51.19% of the total.

##### 3.2.3.1 Social/professional role and identity

As a medical personnel, their sense of responsibility to patients urges them to do a good job of Internet diagnosis and treatment. More than 60% of the participants indicated that the obstacles (such as distance and cost) in the offline diagnosis and treatment of patients were the reason why they were willing to participate in the Internet hospital. It can be seen that the professional attitude of medical personnel hoping to better serve patients would also prompt them to participate in the Internet hospital.

*“I have a sense of responsibility and care for the patients in the Internet hospital.”*

##### 3.2.3.2 Beliefs about capabilities

The limited time, the higher requirements for diagnostic ability, and the uncertainty caused by the lack of relevant examinations all may make doctors question whether they can effectively serve patients. This lack of self-efficacy can hinder their use of Internet hospitals (22, 23).

*"The psychological experience of doctors in online diagnosis and treatment is different from that offline: Online consultation is relatively uncertain so I may feel unsure about my diagnosis.”*

##### 3.2.3.3 Optimism

Medical personnel who have had positive experiences with Internet consultations as patients tend to be optimistic about Internet medical treatment. However, medical risks due to the lack of adequate legal safeguards and low accuracy in Internet diagnosis reduce medical personnel confidence in achieving their goals.

*"Personally, I made inquiries in some public accounts and pay different prices for different doctors, they all listened carefully and answered questions in detail, rather than copied and pasted the textbook for you, so that you feel the money is well spent.”*

*“At present, the rate of misdiagnosis and missed diagnosis on the Internet consultation is high.”*

##### 3.2.3.4 Beliefs about consequences

Unlike the “Optimism” domain which emphasizes confidence in achieving the best possible outcome, this domain focuses on the participants' prediction of a variety of possible outcomes. Affected by different realistic factors, medical personnel will have different expectations of results, which will have different impacts on their participation.

Internet hospitals use digital technology for accessible and convenient medical services, promoting follow-up visits and patient compliance. Participants noted social benefits of Internet hospital applications for hospitals, such as image and popularity is also an enabler. However, diagnostic difficulties including the absence of physical examination, incomplete history, bias in patient narratives and discontinuity in communication may lead to inaccurate diagnoses. Inadequate legal and institutional protection may harm doctor-patient relationships, hindering medical personnel participation.

*"Avoid some face to face embarrassment (for example, some diseases are difficult for patients to talk about), reduce traffic jams around the hospital, and avoid unnecessary second visit to the hospital for prescribing the same medicine.”*

*"It is not conducive to physical examination and family guidance, because rehabilitation professional needs a lot of physical examination, and the family guidance (of rehabilitation movements) sometimes need to be taught face-to-face.”*

##### 3.2.3.5 Reinforcement

Some participants expressed their disagreements of lack of economic incentives because free use may bring a waste of resources, but also make oneself lack of motivation as it occupies non-working time. The low diagnostic accuracy in past practice may also reduce their probability to participate in the Internet hospital (24).

*“Not charging allows some people to waste resources excessively.”*

##### 3.2.3.6 Intentions

Participants expressed a intention to be rewarded financially for their extra workload. The lack of an appropriate reward may hinder their decision to participate in Internet care.

*“Internet diagnosis and treatment takes more time and should be paid economically.”*

The use of Internet hospitals can promote effective diagnosis and treatment from the aspects of return visit, follow-up and patient tracking. The actual needs of medical personnel in these areas will increase their propensity to participate in Internet hospitals.

*“I can know the situation of patients in advance, which is conducive to the return visit and save time.”*

##### 3.2.3.7 Goals

“Goals” domain indicated the importance of Internet hospital to participants. Some participants placed Internet hospital as a low priority due to factors such as time, diagnostic accuracy and risk, but others saw it as a better achieving the goal of whole-process medical treatment which would facilitate their participation.

*“Internet hospitals have been basically suspended due to excessive use of personal time, inefficient communication and high medical risks.”*

*“Using the Internet makes it easy to answer patient consultations and follow up regularly.”*

##### 3.2.3.8 Emotion

Internet therapy will bring different emotional responses and feelings than offline therapy. As we mentioned in “2.3.2 Beliefs about capabilities”, the uncertainty of online counseling can leave doctors feeling uncertain. Hesitation is one of the emotional barrier factors of Internet hospital promotion.

Internet hospital consultation mostly stays in text communication, and the lack of face-to-face communication make it difficult to build trust between doctors and patients, which is another obstacle mentioned by participants.

*"The process of communication lacks emotional support, and it is difficult to establish a sense of trust between doctors and patients in a short time.”*

The ten most frequently mentioned factors are summarised in Table 4 (17).

**Table 4:** Summary of the ten most frequently mentioned factors.

Barriers	
2. Skills (An ability or proficiency acquired through practice)	Diagnostic capability: Put forward higher requirements for doctors’ diagnostic ability
10. Memory, attention and decision processes (The ability to retain information, focus selectively on aspects of the environment and choose between two or more alternatives)	Difficulties in decision process
11. Environmental context and resources (Any circumstance of a person's situation or environment that discourages or encourages the development of skills and abilities, independence, social competence and adaptive behaviour)	Limited resources
	Not ideal setup
4. Beliefs about capabilities (Acceptance of the truth, reality or validity about an ability, talent or facility that a person can put to constructive use)	Lack of self-efficacy
5. Optimism (The confidence that things will happen for the best or that desired goals will be attained): The confidence of medical personnel in the outcomes that can be achieved by participating in Internet hospitals.	Medical risk and low diagnostic accuracy in past practice
6. Beliefs about consequences (Acceptance of the truth, reality, or validity about outcomes of a behaviour in a given situation): The doctors’ opinion about what could happen from performing Internet diagnosis and treatment.	Negative outcome expectation: Limited effectiveness and low accuracy in diagnosis
Enablers	
12. Social influences (Those interpersonal processes that can cause individuals to change their thoughts, feelings, or behaviours)	Patients’ preferences and needs
6. Beliefs about consequences (Acceptance of the truth, reality, or validity about outcomes of a behaviour in a given situation): The doctors’ opinion about what could happen from performing Internet diagnosis and treatment.	Positive outcome expectations in the full medical processes, patients’ experience impression, and hospital social benefits
8. Intentions (A conscious decision to perform a behaviour or a resolve to act in a certain way)	The use of Internet hospitals can promote effective diagnosis and treatment

## 4 Discussion

This study aims to understand the factors influencing the participation and use of Internet hospitals. By mapping the data to the TDF and COM-B frameworks, we identify and demonstrate the challenges and positive factors associated with current Internet hospital participation. Through subject induction and COM-B framework analysis, we provide a comprehensive conceptualization of our findings, which serve as the basis for future behavioral intervention practices (21).

The results show that all theoretical fields have an impact on Internet hospital practice. While barriers are mentioned more frequently than enablers, medical staff generally hold a positive attitude towards the implementation and use of Internet hospitals. Among the seven capability themes, digital health knowledge, training, skills, and communication ability have been previously mentioned in the literature as important for medical personnel (25–38). Regarding opportunity, time constraints, and inefficiencies resulting from network or operating system imperfections discourage healthcare personnel from participating, aligning with past literature (21, 25, 27, 30, 39–44). Motivation, mentioned most frequently, is identified as the main factor influencing medical personnel's participation in Internet hospitals. Five theoretical areas, including beliefs about consequences, skills, decision processes, environmental context, and resources, strongly influence Internet hospital participation. These most frequently cited factors and the intervention types they require are discussed below.

### 4.1 Key factors analysis and implications for practice

#### 4.1.1 Beliefs about consequences and capabilities

In the research study, numerous healthcare professionals have indicated that the implementation of Internet-based healthcare services holds substantial potential in improving the delivery of medical care. Despite their enthusiasm towards their profession and the positive implications of Internet hospitals, medical personnel may face obstacles that undermine the aforementioned motivations, such as a lack of self-efficacy and negative outcome expectations (21, 27).

Online consultations present distinct challenges in comparison to traditional face-to-face interactions, including limitations in conducting physical examinations and imaging procedures, as well as reduced opportunities for direct communication and observation. These disparities can impact the accessibility of crucial diagnostic information, thereby influencing medical personnel's confidence in their own capabilities and their expectations regarding the reliability of online diagnosis results.

To address these barriers and promote Internet hospital participation, interventions such as enablement, education, and training are crucial (45). Investing in advanced diagnostic tools and AI-enabled decision support systems can enhance the accuracy and effectiveness of online consultations (46). These technologies provide valuable support to medical personnel, increasing their confidence and improving patient outcomes. Additionally, educational and training programs should focus on enhancing medical personnel's self-efficacy in using telemedicine platforms, specifically in remote patient assessment, communication and online record keeping (47). By equipping medical professionals with the necessary knowledge and skills will encourage their active participation in Internet hospital practices (25).

#### 4.1.2 Skills and decision-process

In this study, the process of influence on the behavior of medical personnel's participation in Internet hospitals demonstrated by these two domains is similar to “Beliefs about consequences and capabilities”. Objective differences in information acquisition, communication, and examination between online consultations and face-to-face diagnosis and treatment lead some medical personnel to question their diagnostic and communication ability to provide Internet diagnosis and treatment services, and also increases the difficulty of the decision-making process. Information access and communication challenges are particularly prominent in pediatric care, as children may struggle to express their feelings accurately and parents may experience heightened worry and anxiety. Overcoming these barriers requires pediatricians to possess both strong professional skills and the ability to guide children effectively during online consultations, placing greater demands on their diagnostic and communication abilities.

Research indicates that experienced professionals are more likely to have positive attitudes towards e-health services (35). To enhance their participation in Internet diagnosis and treatment, doctors should continually adapt and identify suitable online methods that align with their personal and disciplinary characteristics. Hospitals can support medical staff by providing targeted and ongoing education and training in Internet diagnosis and treatment, promoting effective communication strategies, and improving their utilization of diagnostic technology (25, 45, 48). This, in turn, enhances their ability to participate in Internet diagnosis and treatment.

#### 4.1.3 Environmental and resources

Medical professionals' participation in Internet hospitals is influenced by external opportunities. However, several barriers affect the effectiveness and acceptability of these hospitals: limited time resources due to heavy workload, technical limitations, and imperfections in the system and legal protection hinder medical staff's involvement. Previous research has also recognized similar challenges, including their free time occupation, and inefficiencies from network or operating system imperfections (21, 25, 27, 30, 39–44). Furthermore, inadequate support mechanisms and insufficient legal protection exacerbate these barriers. Medical professionals may hesitate to participate due to concerns about liability and responsibility in online healthcare services.

To promote active engagement in Internet hospitals, three key areas must be prioritized (45). Firstly, upgrading the technical infrastructure through hardware and software improvements is essential for a seamless online healthcare experience. Secondly, comprehensive training programs and ongoing technical support should be provided to address capability and motivation. Lastly, establishing clear guidelines and regulations that define the rights, responsibilities, and liabilities of medical professionals in online healthcare services will instill confidence, address concerns, mitigate legal risks, and promote active involvement in Internet hospitals.

### 4.2 Strengths and limitations

There are limitations to consider in this study, such as the use of a single healthcare organization and a focus on specific aspects of online consultation in an Internet hospital. These factors may restrict the generalizability of the findings to other healthcare populations and digital health systems. A relatively small sample size may be attributed to the study coinciding with the local peak of Omicron secondary infections, resulting in limited availability of medical personnel, but the findings we present are nonetheless insightful and contribute significantly to the current understanding of the perceptions of telemedicine and Internet hospital technologies among medical personnel. Nevertheless, participants varied across a range of variables, including age, years of practice, department, experience with Internet hospitals etc., and thematic saturation was achieved, indicating the results are comprehensive in coverage of the issues (49).

The use of the TDF and COM-B model is a notable strength of this study. These frameworks provide a comprehensive analysis of factors influencing behavior and identify each influence factor through component within each domain. The study identified numerous enablers and barriers, thereby proposing a comprehensive framework of enablers and barriers for the utilization of Internet hospitals, increasing confidence in the effectiveness of the TDF and COM-B model in assessing behavioral problems (50). By combining COM-B with BCW and BCT, interventions can be tailored and effective optimization measures can be formulated. While these behavior change frameworks have been employed in various clinical practices, their application in Internet hospital consultation is relatively new (51). The TDF domains faced challenges regarding definition ambiguity and domain overlap, which may impact consensus, but this study achieved a high level of agreement in coding-related barriers and enabling factors (52).

So despite these limitations, the study offers valuable insights into the factors influencing the engagement of medical personnel in Internet hospitals. The ultimate goal of a series of studies is to optimize the construction of Internet hospitals, necessitating further intervention measures. As a first step, the research served as an exploratory study, generating hypotheses rather than testing them, and identified which areas for the development of Internet hospitals. The subsequent step involves identifying the most crucial factors impacting behavior and utilizing existing results to inform the matching of interventions and behavioral change techniques, thereby facilitating the formulation of detailed optimization measures using BCT. Extending the study to encompass broader healthcare organizations and digital health systems would confirm and enhance the generalizability of these findings, or to explore the impact of infrastructure construction in different medical environments on the attitudes of medical workers towards Internet hospitals is also a viable idea for further research (49).

## 5 Conclusion

Currently, the Internet hospital is acknowledged to have advantages s they promote effective medical treatment and optimize the allocation of medical resources, but its development also faces problems and challenges. This qualitative study explored the barriers and enablers influencing medical personnel's participation in Internet hospitals using the TDF and COM-B frameworks from the perspective of medical personnel and motivation emerged as key factors. While barriers outweighed enablers, attitudes towards Internet hospitals were generally optimistic.

These findings provide valuable insights into optimizing Internet hospital implementation through targeted interventions such as training and technology optimization. Additionally, the TDF and COM-B analyses enrich the understanding of individual-level factors to include wider contextual drivers, contributing a conceptual framework for understanding multi-level influences on digital health behavior change. However, future research should validate these findings across larger and more diverse healthcare systems due to the single-center design, to develop tailored implementation strategies. With targeted optimization informed by behavioral science models, Internet hospitals are expected to be more adopted and their benefits for improving medical services will be increased.

## Data Availability

The original contributions presented in the study are included in the article/Supplementary Material, further inquiries can be directed to the corresponding author.
