# Supplementary material for: Analyzing the barriers and enablers to internet hospital implementation: a qualitative study of a tertiary hospital using TDF and COM-B framework
Source: Front Digit Health. 2024 Aug 8;6:1362395. doi: 10.3389/fdgth.2024.1362395 (PMC11340510; doi:10.3389/fdgth.2024.1362395)
Supplement: Supplementary file 1 [file Datasheet1.docx]

**Table** **1. The questionnaire outline**

| **Attributes** | **Topics** | |
| --- | --- | --- |
| Personal Information | Name, age, department, specialties, years of working, professional title | |
| Whether to provide relevant services/use relevant technology/carry out relevant work and how | Yes | 1. How to obtain it  2. The reasons & How to organize and promote  3. The process  4. Personal Specific tasks & How was it accomplished & Memorable experiences  5. The degree of difficulty to provide/use/carry out |
|  | No | 1. The intended time to do  2. The preparations |
| Significance/Goals | 1. The opinions on the effectiveness of Internet hospitals  2. Whether it worked to achieve your goals and why | |
| Negative impacts | \ | |
| Comparative advantages and disadvantages with traditional modes | \ | |
| Similarities and differences with traditional modes | \ | |
| The influencing factors of the implementation | Capabilities | What capabilities are needed (knowledge/skills/psychological capabilities) |
|  | Opportunities | 1. The impacts of time and resources  2. How to view the technology itself and the usage process  3. How to use it in contact with objects  4. How others influence the practice process |
|  | Motivations | 1. The feelings about using it or not using it  2. Do you consider using it beneficial and why |
| How to evaluate your performance | \ | |
| Current difficulties | 1. The solutions you can think of  2. What resources and support could assist you | |
