# Supplementary material for: Analyzing the barriers and enablers to internet hospital implementation: a qualitative study of a tertiary hospital using TDF and COM-B framework
Source: Front Digit Health. 2024 Aug 8;6:1362395. doi: 10.3389/fdgth.2024.1362395 (PMC11340510; doi:10.3389/fdgth.2024.1362395)
Supplement: Supplementary file 2 [file Datasheet2.docx]

**Table** **2. Summary of participants' characteristics**

| **Age (mean: 37.24)** | |
| --- | --- |
| 20-30 | 4 |
| 31-40 | 14 |
| 41-50 | 5 |
| 51-60 | 2 |
| **Years of practice (mean: 12.68)** | |
| 0-10 | 11 |
| 11-20 | 11 |
| >20 | 3 |
| **Category of Department** | |
| Clinical departments (18) | Obstetrics (4/18) |
|  | Pediatrics (5/18) |
|  | Otolaryngology (1/18) |
|  | Gynecology (1/18) |
|  | Emergency (1/18) |
|  | Reproductive Immunology (1/18) |
|  | Nutrition (1/18) |
|  | Acupuncture and Tuina (1/18) |
|  | Traditional Chinese Medicine (2/18) |
|  | Dermatology (1/18) |
| Health Care Department (3) | Children's Psychology and Rehabilitation (2/3) |
|  | Women's Health (1/3) |
| Medical Technology Department (3) | Clinical Laboratory (1/3) |
|  | Pathology (1/3) |
|  | Ultrasound (1/3) |
| Administrative Department (1) | Administrative Department (1/1) |
| **Professional title** | |
| Professor of Medicine | 3 |
| Associate doctor | 5 |
| Attending doctor | 10 |
| Resident doctor | 3 |
| Deputy nurse-in-charge | 1 |
| Nurse-in-charge | 3 |
| **Whether to participate in the Internet online consultation platform** | |
| Yes | 13 |
| No | 12 |
