# Supplementary material for: Analyzing the barriers and enablers to internet hospital implementation: a qualitative study of a tertiary hospital using TDF and COM-B framework
Source: Front Digit Health. 2024 Aug 8;6:1362395. doi: 10.3389/fdgth.2024.1362395 (PMC11340510; doi:10.3389/fdgth.2024.1362395)
Supplement: Supplementary file 3 [file Datasheet3.docx]

**Table** **3. Table of findings by TDF domains and COM-B model[17][20]**

| **Domains** | **Themes (Barriers/Enablers)** | **References** |
| --- | --- | --- |
| **Capability (34.00%)** | | |
| 1. Knowledge: What doctors and patients know on Internet hospital diagnosis and treatment. | Doctors' affirmative understanding of Internet hospital practice (E) | "The Internet hospital is a progressive product that can facilitate patients' treatment……" |
|  | Patients' limited exposure/knowledge of eHealth (B) | More than half of the participants said that patients' view that in-person medical care is superior to telemedicine is an important reason that hinders medical staff from participating in Internet hospital diagnosis and treatment. |
| 2. Skills: What doctors know about how they should perform on the Internet hospital. | Diagnostic capability: Put forward higher requirements for doctors' diagnostic ability (B) | "I need to complete the diagnosis and treatment tasks independently without the guidance of a superior doctor."  "Because we can't diagnosis face to face, we need to make a treatment plan under the situation that the medical history and examination are not very clear." |
|  | Interpersonal skills: Put forward higher requirements for doctors' interpersonal and communication skills (B) | "Online diagnosis and treatment is mostly discontinuous, the delay time is long, the family expression is often lengthy, consuming the patience of medical staff." |
|  | Technical proficiency and relevant training (B) | "Poor application of technology will affect work efficiency." |
| 10. Memory, attention and decision processes: Decision process regarding Internet hospital diagnosis and treatment. | Difficulties in decision process (B) | "Due to resource constraints and inability to check the body, there is an evaluation bias." |
| 14. Behavioural regulation: Management of doctors' diagnosis and treatment behavior in Internet hospitals. | Lack of clear treatment guidelines (B) | "At present, there is a lack of specific guidelines for Internet hospital diagnosis and treatment." |
| **Opportunity (14.81%)** | | |
| 11. Environmental context and resources: Influence of the environment on doctors’ behaviour. | Limited resources (B) | "Doctors have limited time and energy, and discontinuous communication can eat into rest time." |
|  | Technical limitation (B) | "Network speed affects the effectiveness of Internet diagnosis and treatment." |
|  | Not ideal setup (B) | "If the Internet hospital system and mechanism is more sound and can effectively protect the safety of doctors, nurses and patients... I support the launch of the Internet hospital." |
| 12. Social influences: How others influence doctors' behaviour of Internet diagnosis and treatment. | Patients' preferences and needs (E) | "It is easier for people to see a doctor and consult a doctor, and they can visit a specialist without limitation by geography." |
|  | Social pressure(B) | "Many parents are prone to anxiety and expect complete satisfaction without seeing the patient." |
| **Motivation (51.19%)** | | |
| 3. Social/professional role and identity: Personal qualities such as professional attitude and responsibility of medical personnel. | Professional Identity/Professional sense of responsibility (E) | "I have a sense of responsibility and care for the patients in the Internet hospital." |
| 4. Beliefs about capabilities: Perceived capability of doctors to perform Internet diagnosis and treatment. | Lack of self-efficacy (B) | "The psychological experience of doctors in online diagnosis and treatment is different from that offline: Online consultation is relatively uncertain so I may feel unsure about my diagnosis." |
| 5. Optimism: The confidence of medical personnel in the outcomes that can be achieved by participating in Internet hospitals. | The doctor's past experience brings confidence (E) | "Personally, I made inquiries in some public accounts and pay different prices for different doctors, they all listened carefully and answered questions in detail, rather than copied and pasted the textbook for you, so that you feel the money is well spent." |
|  | Medical risk and low diagnostic accuracy in past practice (B) | "At present, the rate of misdiagnosis and missed diagnosis on the Internet consultation is high." |
| 6. Beliefs about Consequences: The doctors' opinion about what could happen from performing Internet diagnosis and treatment. | Positive outcome expectations in the full medical processes, patients' experience impression, and hospital social benefits (E) | "Avoid some face to face embarrassment (for example, some diseases are difficult for patients to talk about), reduce traffic jams around the hospital, and avoid unnecessary second visit to the hospital for prescribing the same medicine." |
|  | Negative outcome expectation: Limited effectiveness and low accuracy in diagnosis (B) | "It is not conducive to physical examination and family guidance, because rehabilitation professional needs a lot of physical examination, and the family guidance (of rehabilitation movements) sometimes need to be taught face-to-face." |
| 7. Reinforcement: Measures to stimulate the willingness of medical personnel to participate. | Lack of economic incentives (B) | "Not charging allows some people to waste resources excessively." |
|  | Low diagnostic accuracy in past practice (B) | "Online diagnosis will not be too accurate, and the rate of misdiagnosis and missed diagnosis is high." |
| 8. Intentions: The inclination of doctors to practice Internet diagnosis and treatment. | Lack of motivation：Added workload and hope for monetary rewards (B) | "Internet diagnosis and treatment takes more time and should be paid economically." |
|  | The use of Internet hospitals can promote effective diagnosis and treatment (E) | "I can know the situation of patients in advance, which is conducive to the return visit and save time." |
| 9. Goals: How important is Internet hospital practice for stakeholders. | Low priority of the Internet hospital (B) | "Internet hospitals have been basically suspended due to excessive use of personal time, inefficient communication and high medical risks." |
|  | Better achieving the goal of whole-process medical treatment (E) | "Using the Internet makes it easy to answer patient consultations and follow up regularly." |
| 13. Emotion: How doctors feel during the Internet hospital process. | Hesitation (B) | "Due to the uncertainty of online consultation, the psychological feelings of doctors in online diagnosis and treatment are different from offline, and they will be mentally uncertain and hesitation." |
|  | Lack of trust between doctors and patients (B) | "The process of communication lacks emotional support, and it is difficult to establish a sense of trust between doctors and patients in a short time." |
