# Supplementary material for: Analyzing the barriers and enablers to internet hospital implementation: a qualitative study of a tertiary hospital using TDF and COM-B framework
Source: Front Digit Health. 2024 Aug 8;6:1362395. doi: 10.3389/fdgth.2024.1362395 (PMC11340510; doi:10.3389/fdgth.2024.1362395)
Supplement: Supplementary file 4 [file Datasheet4.docx]

Table 4. Summary of the ten most frequently mentioned factors

| **Barriers** | |
| --- | --- |
| 2. Skills  (An ability or proficiency acquired through practice) | Diagnostic capability: Put forward higher requirements for doctors' diagnostic ability |
| 10. Memory, attention and decision processes  (The ability to retain information, focus selectively on aspects of the environment and choose between two or more alternatives) | Difficulties in decision process |
| 11. Environmental context and resources  (Any circumstance of a person’s situation or environment that discourages or encourages the development of skills and abilities, independence, social competence and adaptive behaviour) | Limited resources |
|  | Not ideal setup |
| 4. Beliefs about capabilities  (Acceptance of the truth, reality or validity about an ability, talent or facility that a person can put to constructive use) | Lack of self-efficacy |
| 5. Optimism  (The confidence that things will happen for the best or that desired goals will be attained): The confidence of medical personnel in the outcomes that can be achieved by participating in Internet hospitals. | Medical risk and low diagnostic accuracy in past practice |
| 6. Beliefs about Consequences  (Acceptance of the truth, reality, or validity about outcomes of a behaviour in a given situation): The doctors' opinion about what could happen from performing Internet diagnosis and treatment. | Negative outcome expectation: Limited effectiveness and low accuracy in diagnosis |
| **Enablers** | |
| 12. Social influences  (Those interpersonal processes that can cause individuals to change their thoughts, feelings, or behaviours) | Patients' preferences and needs |
| 6. Beliefs about Consequences  (Acceptance of the truth, reality, or validity about outcomes of a behaviour in a given situation): The doctors' opinion about what could happen from performing Internet diagnosis and treatment. | Positive outcome expectations in the full medical processes, patients' experience impression, and hospital social benefits |
| 8. Intentions  (A conscious decision to perform a behaviour or a resolve to act in a certain way) | The use of Internet hospitals can promote effective diagnosis and treatment |
