# Supplementary material for: Analyzing the barriers and enablers to internet hospital implementation: a qualitative study of a tertiary hospital using TDF and COM-B framework
Source: Front Digit Health. 2024 Aug 8;6:1362395. doi: 10.3389/fdgth.2024.1362395 (PMC11340510; doi:10.3389/fdgth.2024.1362395)
Supplement: Supplementary file 5 [file Datasheet5.docx]

Additional File 1: COREQ checklist

Developed from: Tong A, Sainsbury P, Craig J. Consolidated criteria for reporting qualitative research (COREQ): a 32-item checklist for interviews and focus groups. International journal for quality in health care. 2007; 19(6):349-57.

| No. Item | Guide questions/description | Reported on Section |
| --- | --- | --- |
| Domain 1: Research team and reﬂexivity | | |
| *Personal Characteristics* |  |  |
| 1. Interviewer/facilitator | Which author/s conducted the interview or focus group? | Declarations-Authors' contributions |
| 2. Credentials | What were the researcher’s credentials? | Declarations-Authors' information |
| 3. Occupation | What was their occupation at the time of the study? | Declarations-Authors' information |
| 4. Gender | Was the researcher male or female? | Not reported |
| 5. Experience and training | What experience or training did the researcher have? | Declarations-Authors' information |
| *Relationship with participants* | | |
| 6. Relationship established | Was a relationship established prior to study commencement? | 2 Methods-2.1 Participants |
| 7. Participant knowledge of the interviewer | What did the participants know about the researcher? | 2 Methods-2.1 Participants |
| 8. Interviewer characteristics | What characteristics were reported about the interviewer/facilitator? | Declarations-Authors' information |
| Domain 2: Study design | | |
| *Theoretical framework* | | |
| 9. Methodological orientation and Theory | What methodological orientation was stated to underpin the study? | 2 Methods |
| *Participant selection* | | |
| 10. Sampling | How were participants selected? | 2 Methods-2.1 Participants |
| 11. Method of approach | How were participants approached? | 2 Methods-2.1 Participants |
| 12. Sample size | How many participants were in the study? | 2 Methods-2.1 Participants |
| 13. Non-participation | How many people refused to participate or dropped out? Reasons? | Not reported |
| *Setting* |  |  |
| 14. Setting of data collection | Where was the data collected | 2 Methods-2.1 Participants |
| 15. Presence of non-participants | Was anyone else present besides the participants and researchers? | Not reported |
| 16. Description of sample | What are the important characteristics of the sample? | 2 Methods-2.1 Participants |
| *Data collection* |  |  |
| 17. Interview guide | Were questions, prompts, guides provided by the authors? Was it pilot tested? | 2 Methods-2.2 Questionnaire Development and Data Analysis |
| 18. Repeat interviews | Were repeat interviews carried out? If yes, how many? | N/A |
| 19. Audio/visual recording | Did the research use audio or visual recording to collect the data? | N/A |
| 20. Field notes | Were ﬁeld notes made during and/or after the interview or focus group? | N/A |
| 21. Duration | What was the duration of the interviews or focus group? | N/A |
| 22. Data saturation | Was data saturation discussed? | 2 Methods-2.3 Data Analysis |
| 23. Transcripts returned | Were transcripts returned to participants for comment and/or correction? | N/A |
| Domain 3: Analysis and ﬁndings | | |
| *Data analysis* | | |
| 24. Number of data coders | How many data coders coded the data? | 3 Results-3.2 Factors influencing Internet hospital implementation |
| 25. Description of the coding tree | Did authors provide a description of the coding tree? | 3 Results-3.2 Factors influencing Internet hospital implementation |
| 26. Derivation of themes | Were themes identiﬁed in advance or derived from the data? | 3 Results-3.2 Factors influencing Internet hospital implementation |
| 27. Software | What software, if applicable, was used to manage the data? | 3 Results-3.2 Factors influencing Internet hospital implementation |
| 28. Participant checking | Did participants provide feedback on the ﬁndings? | Not reported |
| *Reporting* |  |  |
| 29. Quotations presented | Were participant quotations presented to illustrate the themes/ﬁndings? Was each quotation identiﬁed? | 3 Results-3.2 Factors influencing Internet hospital implementation |
| 30. Data and ﬁndings consistent | Was there consistency between the data presented and the ﬁndings? | 4 Discussion |
| 31. Clarity of major themes | Were major themes clearly presented in the ﬁndings? | 4 Discussion |
| 32. Clarity of minor themes | Is there a description of diverse cases or discussion of minor themes? | N/A |
